# DPF–EHDNet: a differential-path and structurally enhanced network for thyroid ultrasound segmentation

**DOI:** 10.3389/fmed.2026.1803455

**Published:** 2026-05-19

**Authors:** Xuefei Feng, Le Su, Yuhao Tian, Chunjie Guo, Wenjun Hu, Yulong Wang, Zihan Lv, Jun Li

**Affiliations:** 1 College of Information Engineering, Sichuan Agricultural University, Ya'an, China; 2 College of Mechanical and Electrical Engineering, Sichuan Agricultural University, Ya'an, China; 3 Agricultural Information Engineering Higher Institution Key Laboratory of Sichuan Province, Ya'an, China; 4 Ya'an Digital Agricultural Engineering Technology Research Center, Ya'an, China

**Keywords:** differential feature modeling, edge-aware decoding, structure-aware segmentation, thyroid ultrasound segmentation, ultrasound image analysis

## Abstract

Accurate segmentation of thyroid nodules in ultrasound remains challenging due to speckle noise, low-contrast lesion margins, heterogeneous echogenicity, and substantial inter-case variability. Reliable delineation is essential for supporting clinical assessment and downstream computer-assisted diagnosis. We propose DPF–EHDNet, a differential-path and structurally enhanced segmentation framework tailored to challenging ultrasound conditions. The network integrates differential-path feature enhancement to suppress speckle-induced ambiguity, edge-aware multi-scale context encoding to preserve anatomical structure, and confidence-guided shallow feature fusion to improve localization consistency. Our model is trained under a fixed-iteration protocol without validation-based checkpoint selection to ensure fair comparison across methods, and its performance is reported as the average over three random seeds. Experiments on a thyroid ultrasound dataset constructed from three publicly available benchmarks (DDTI, TN-SCUI2020, and TN3K) show that DPF–EHDNet achieves 93.09% mIoU, 93.35% Dice, 93.98% precision, and 92.73% recall, consistently outperforming representative baseline segmentation approaches. DPF–EHDNet provides a robust and structurally consistent solution for thyroid ultrasound segmentation under speckle degradation and low-contrast conditions, demonstrating its potential to support clinical workflows and computer-assisted ultrasound analysis.

## 1 Introduction

Thyroid nodules are among the most common abnormalities detected in routine ultrasound examinations and play a critical role in early diagnosis and malignancy risk assessment of thyroid diseases (1, 2). Ultrasound is widely adopted in clinical practice due to its safety, accessibility, and real-time imaging capability. However, diagnostic outcomes remain highly operator-dependent. Variations in probe pressure, scanning angle, and subjective interpretation, together with modality-specific artifacts such as speckle noise, heterogeneous echogenicity, acoustic shadowing, and low lesion–background contrast, pose persistent challenges to reliable thyroid nodule segmentation (3).

Deep learning–based segmentation frameworks, including U-Net (4), DeepLab (5), and nnU-Net (6), have achieved remarkable success in computed tomography, magnetic resonance imaging, and other medical imaging modalities. Nevertheless, ultrasound segmentation remains substantially more challenging. Speckle noise disrupts local texture patterns, iso-echoic nodules exhibit weak intensity contrast, and structural appearance varies considerably across patients and acquisition settings. These factors often cause conventional convolutional neural networks to produce over-smoothed boundaries or unstable segmentation results, limiting their robustness in real-world thyroid ultrasound scenarios.

Recent advances have explored the integration of foundation models and Transformer-based architectures for medical image segmentation. In particular, the Segment Anything Model (SAM) and its variants have been adapted to ultrasound imaging tasks, demonstrating promising generalization capabilities in breast lesion segmentation. For example, a dual-branch SAM-Transformer fusion network has been proposed to improve segmentation accuracy in ultrasound images (7), while another study investigated the applicability of SAM for breast tumor detection (8). Furthermore, a SAM adapter tailored for ultrasound data was introduced to enable better adaptation to domain-specific characteristics such as speckle noise and low contrast (9).

Despite these advances, existing SAM-based and Transformer-driven approaches often rely on large-scale pretraining and global attention mechanisms, which may not fully capture fine-grained structural variations and boundary details in thyroid ultrasound images. In contrast, accurate delineation of thyroid nodules requires precise modeling of local structural transitions and boundary consistency under noisy imaging conditions.

To mitigate these limitations, existing studies have explored attention mechanisms (10), multi-scale feature aggregation (11), and structure-aware enhancement strategies (12). Although such approaches provide incremental improvements, many rely on global pooling operations or generic attention weighting (13, 14), which are insufficient to capture the complex interaction between contextual structure and noise-corrupted local patterns in thyroid ultrasound images. Consequently, segmentation performance often degrades under severe speckle interference or in the presence of heterogeneous glandular backgrounds.

From a clinical perspective, an effective thyroid ultrasound segmentation framework should enhance discriminative representations while maintaining structural consistency and decoding stability, thereby supporting reliable lesion delineation for downstream diagnosis and risk assessment. This requirement motivates the joint modeling of differential feature responses, multi-scale contextual information, and selective utilization of reliable shallow features, which remain insufficiently addressed in existing methods.

To this end, we propose **DPF–EHDNet**, a thyroid-oriented ultrasound segmentation framework designed to improve structural representation and robustness under challenging imaging conditions. Given the large variability in nodule size and morphology across patients, effective multi-scale contextual modeling is essential for robust segmentation. Accordingly, the proposed framework integrates three complementary components: (1) a *Dual-Path Differential Feature (DPF)* module that explicitly models high–low frequency discrepancies to enhance boundary-sensitive structural responses under speckle interference; (2) an *ASPP-E* module that aggregates multi-scale contextual information while reinforcing structure-aware representations; and (3) an *Edge-aware Hybrid Decoder (EHD)* that employs confidence-guided feature fusion to selectively retain reliable shallow information during the decoding process. Together, these components address ultrasound-specific degradation patterns that are closely associated with diagnostic ambiguity in clinical thyroid ultrasound examinations.

To evaluate robustness and generalizability, we construct a hybrid thyroid ultrasound dataset by integrating multiple publicly available benchmarks with complementary characteristics and acquisition conditions. Extensive experimental results demonstrate that **DPF–EHDNet** consistently outperforms representative baseline models across diverse imaging scenarios, highlighting its effectiveness and stability. These findings indicate that the proposed framework provides a reliable solution for automated thyroid ultrasound segmentation and offers practical potential for computer-assisted clinical assessment.

## 2 Data set and materials

### 2.1 Public thyroid ultrasound datasets

This study employs a hybrid thyroid ultrasound dataset constructed by integrating three publicly available benchmark datasets: DDTI (15), TN-SCUI2020 (16), and TN3K (17). These datasets provide complementary imaging characteristics in terms of acquisition devices, annotation protocols, and nodule appearance, enabling a comprehensive evaluation of segmentation robustness and generalization.

The Digital Database for Thyroid Images (DDTI) (15) contains ultrasound images acquired under diverse clinical settings, exhibiting substantial variations in echogenicity, speckle patterns, and lesion morphology. Owing to its heterogeneity, DDTI has been widely adopted as a benchmark for evaluating thyroid ultrasound segmentation methods.

The TN-SCUI2020 dataset (16) provides high-quality thyroid ultrasound images with expert-annotated nodule boundaries, with particular emphasis on challenging cases characterized by low contrast and heterogeneous internal structures. Its precise annotations make it especially suitable for assessing boundary-sensitive segmentation performance.

The TN3K dataset (17) is a large-scale publicly available thyroid ultrasound benchmark comprising thousands of annotated images collected across multiple ultrasound devices and acquisition conditions. Its scale and diversity support robust evaluation of model performance and generalization under real-world clinical variability.

After data harmonization and quality inspection, all images were resized to a unified spatial resolution and merged into a single hybrid dataset.

The final dataset consists of 3,644 images from TN-SCUI2020, 3,493 images from TN3K, and 470 images from DDTI, resulting in a total of 7,607 images, as summarized in Table 1.

**Table 1:** Statistics of the hybrid thyroid ultrasound dataset used in this study.

Dataset	Number of images	Split strategy
TN-SCUI2020	3,644	Random train/test split
TN3K	3,493	Official train/test split
DDTI	470	Patient-level train/test split
Total	7,607	Overall ratio ≈ 7:3

For TN3K, we directly adopted its official training and testing split to ensure consistency with prior studies. For DDTI, the data were partitioned at the patient level. Specifically, multiple images belonging to the same patient (e.g., 1_1 and 1_2) were assigned to the same subset to avoid data leakage. TN-SCUI2020 contains one image per patient and was therefore divided normally into training and testing subsets.

Overall, the hybrid dataset was organized into training and testing sets with an approximate ratio of 7:3, while ensuring that no patient appeared in both subsets.

### 2.2 Annotation protocol

All datasets provide pixel-level annotations of thyroid nodules. For each dataset, the original ground-truth masks released by the dataset authors were directly adopted. To ensure annotation reliability, all masks were visually inspected for boundary alignment and anatomical plausibility. Samples with obvious inconsistencies or severe annotation errors were excluded from the training set.

### 2.3 Data preprocessing

To reduce variability across imaging devices and acquisition settings, all images were processed using a standardized preprocessing pipeline, which included the following steps:

- **Resolution normalization:** All images and corresponding masks were resized to 256 × 256 pixels.
- **Channel formatting:** Images were retained in single-channel grayscale format, consistent with the physical characteristics of ultrasound acquisition.
- **Intensity normalization:** Pixel intensities were linearly scaled to the [0, 1] range to improve numerical stability during network training.

These preprocessing steps ensure consistent input characteristics across heterogeneous imaging sources and facilitate stable model optimization.

### 2.4 Datasets for generalization experiments

To further assess the cross-domain robustness of the proposed DPF–EHDNet architecture, three additional publicly available datasets were incorporated for generalization experiments:

- **COCO 2017 subset:** A large-scale natural image dataset used to evaluate out-of-domain generalization beyond medical imaging (18).
- **Brain MRI segmentation dataset:** A dataset acquired using a fundamentally different imaging modality, enabling evaluation of cross-modality robustness (19).
- **Breast ultrasound dataset:** A clinically relevant ultrasound dataset containing benign and malignant breast lesions, sharing modality-specific challenges such as speckle noise, low contrast, and boundary ambiguity (20).

These datasets were used for independent training and evaluation following their respective official training and test splits. Together, they enable a systematic assessment of architectural robustness across different imaging modalities, anatomical regions, and data distributions.

## 3 Methods

### 3.1 Overview and motivation

Thyroid ultrasound imaging is inherently affected by modality-specific degradations, including speckle noise, heterogeneous echogenicity, iso-echoic nodules, and low lesion–background contrast. These characteristics pose significant challenges for automated segmentation and frequently lead to unstable or structurally inconsistent predictions when conventional encoder–decoder architectures are applied under diverse clinical acquisition conditions.

In practical thyroid ultrasound segmentation scenarios, three representative degradation patterns can be observed. First, the differential contrast between thyroid nodules and surrounding parenchyma is often weak, particularly in iso-echoic cases. Standard convolutional operations tend to average responses across frequency components, which may suppress subtle but diagnostically relevant structural variations and result in ambiguous feature separability. Second, although atrous spatial pyramid pooling (ASPP) (5) enables multi-scale contextual modeling, it is not explicitly designed to address ultrasound-specific structural ambiguity. Under speckle interference and acoustic shadowing, informative structural cues may be attenuated, leading to over-smoothed segmentation outputs and reduced consistency in representing irregular lesion morphology across scales. Third, shallow encoder features preserve fine spatial details but are highly susceptible to noise and local artifacts. Direct skip connections may therefore introduce noise-dominated activations into the decoding stage, destabilizing feature fusion in low-contrast regions.

To explicitly address these degradation patterns, we propose **DPF–EHDNet**, a DeepLab-style encoder–decoder architecture augmented with three thyroid-oriented modules. The **Dual-Path Differential Feature (DPF)** module is designed to enhance discriminative structural responses by separating detail-related and context-related information, thereby improving sensitivity to weak differential contrast. The **ASPP-E** module extends conventional ASPP by aggregating multi-scale semantic context while reinforcing structure-aware representations under heterogeneous imaging conditions. Furthermore, the **Edge-aware Hybrid Decoder (EHD)** employs confidence-guided feature fusion to selectively retain reliable shallow features during decoding, mitigating the adverse impact of noise-corrupted activations. Each module targets a distinct yet complementary degradation mechanism, including contrast ambiguity, multi-scale structural inconsistency, and unstable feature fusion.

An overview of the proposed DPF–EHDNet architecture and its major components is illustrated in Figure 2.

**Figure 1:**
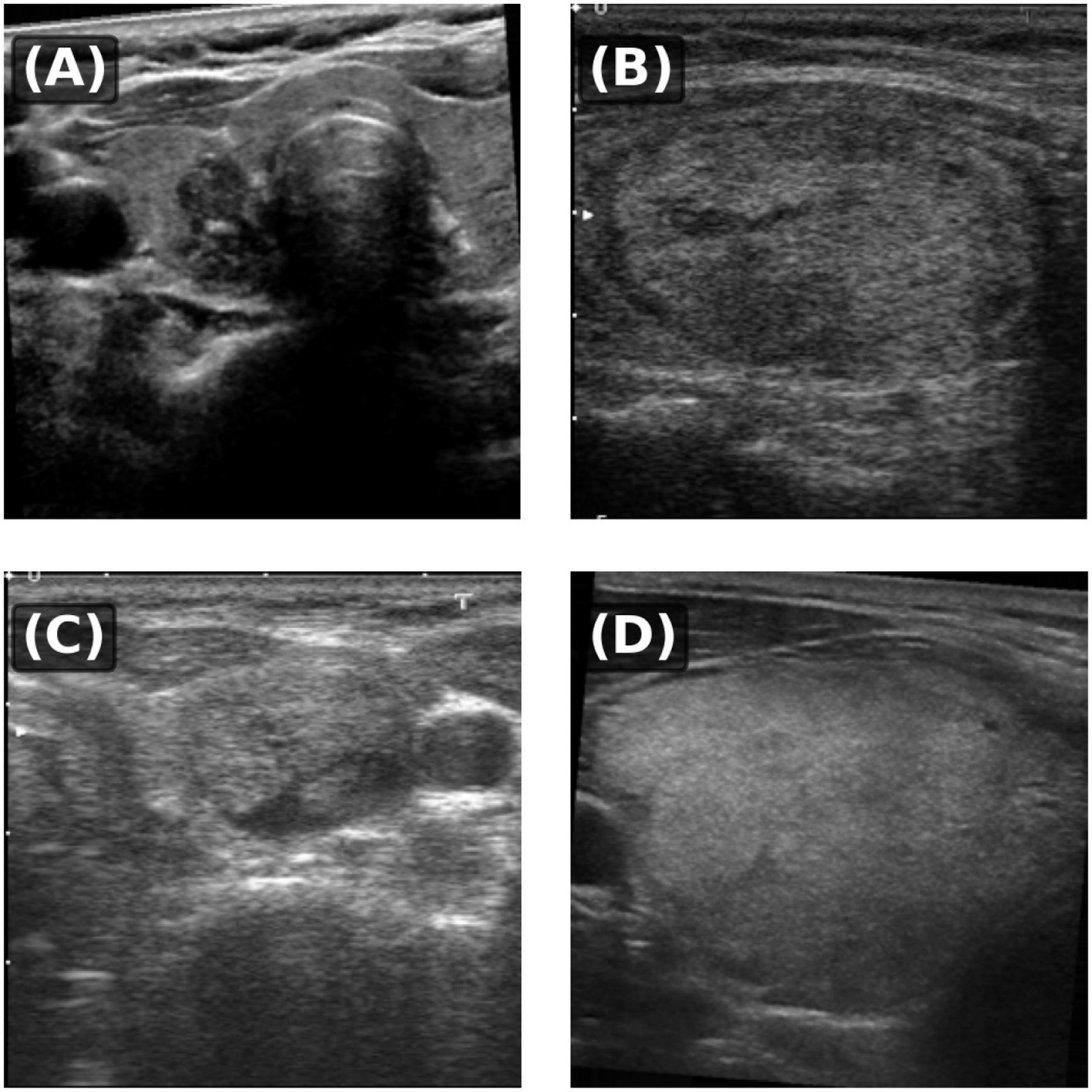
Representative ultrasound images from the curated thyroid dataset: **(A)** a clearly defined nodule with regular margins; **(B)** a low-contrast lesion with blurred and indistinct boundaries; **(C)** a structurally complex case with heterogeneous echogenicity; **(D)** a large homogeneous nodule with weak or missing boundary cues. These examples illustrate the wide variability in thyroid nodule appearance, including irregular boundaries, intensity inhomogeneity, and indistinct lesion margins.

**Figure 2:**
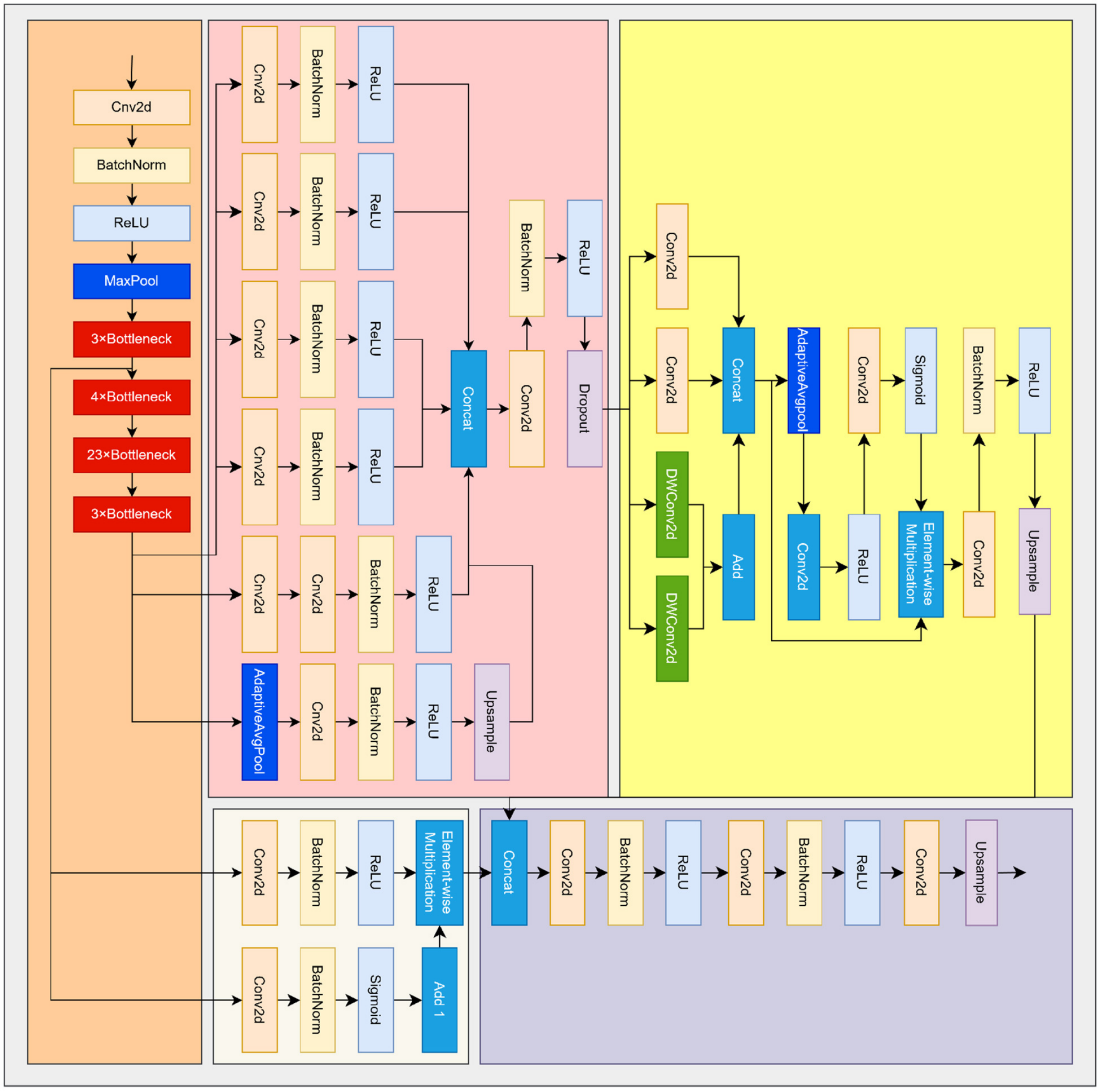
Overall architecture of the proposed DPF–EHDNet. The network adopts a DeepLab-style encoder-decoder backbone and integrates three thyroid-oriented modules: (1) the Dual-Path Differential Feature (DPF) module enhances discriminative structural responses by learnable high- and low-frequency decomposition; (2) the Edge-aware Atrous Spatial Pyramid Pooling with Enhancement (ASPP-E) module aggregates multi-scale semantic context while preserving structurally relevant cues; and (3) the Edge-Guided Hybrid Decoder (EHD) selectively fuses reliable shallow features using an edge-derived confidence map. Together, these components improve robustness to speckle noise, heterogeneous echogenicity, and structural ambiguity commonly encountered in thyroid ultrasound imaging.

### 3.2 Training protocol and hyperparameter settings

All experiments were conducted under a unified training protocol to ensure fair and reproducible comparisons. The backbone network is based on DeepLabV3+ with a ResNet101 encoder, and all models were trained from scratch without using any pre-trained weights. Network parameters were optimized using stochastic gradient descent (SGD) with a momentum of 0.9 and a weight decay of 1 × 10^−4^. The initial learning rate was set to 0.01 and decayed following a polynomial schedule with a power of 0.9. Consistent with the standard DeepLab training strategy, different learning rates were assigned to different network components, with the backbone learning rate fixed at 0.1 times that of the classifier.

Models were trained with a batch size of 8, and all input images were resized to 256 × 256 pixels. Data augmentation was applied exclusively to the training set and consisted of random horizontal flipping. During inference, a batch size of 8 was used. For thyroid ultrasound images, test-time augmentation based on horizontal flipping was adopted, and final predictions were obtained by averaging the corresponding probability maps.

The loss function combined pixel-wise cross-entropy loss with region-based overlap losses (Dice loss and Jaccard loss, implemented in a unified formulation) (6). The weighting coefficient for the cross-entropy term was set to 0.3, while the remaining weight (0.7) was assigned to the overlap loss. Online hard example mining (OHEM) was applied to the cross-entropy term to emphasize hard-to-classify pixels and alleviate class imbalance.

To analyze the robustness of the proposed framework under stochastic optimization, an additional multi-run evaluation was performed by repeating the training process of DPF–EHDNet using three different random seeds. In these experiments, the training–testing split, network architecture, and all hyperparameter settings were kept identical, and the final models after convergence were used for evaluation.

No separate validation set was used in this study. Instead, model selection was based on training convergence, and the use of multiple random seeds further ensured stable and reliable evaluation.

All remaining experiments, including baseline comparisons and ablation studies, were conducted using a single fixed random seed, following common practice to control computational cost while maintaining consistent training conditions.

### 3.3 Dual-path differential feature module (DPF)

**Figure 3:**
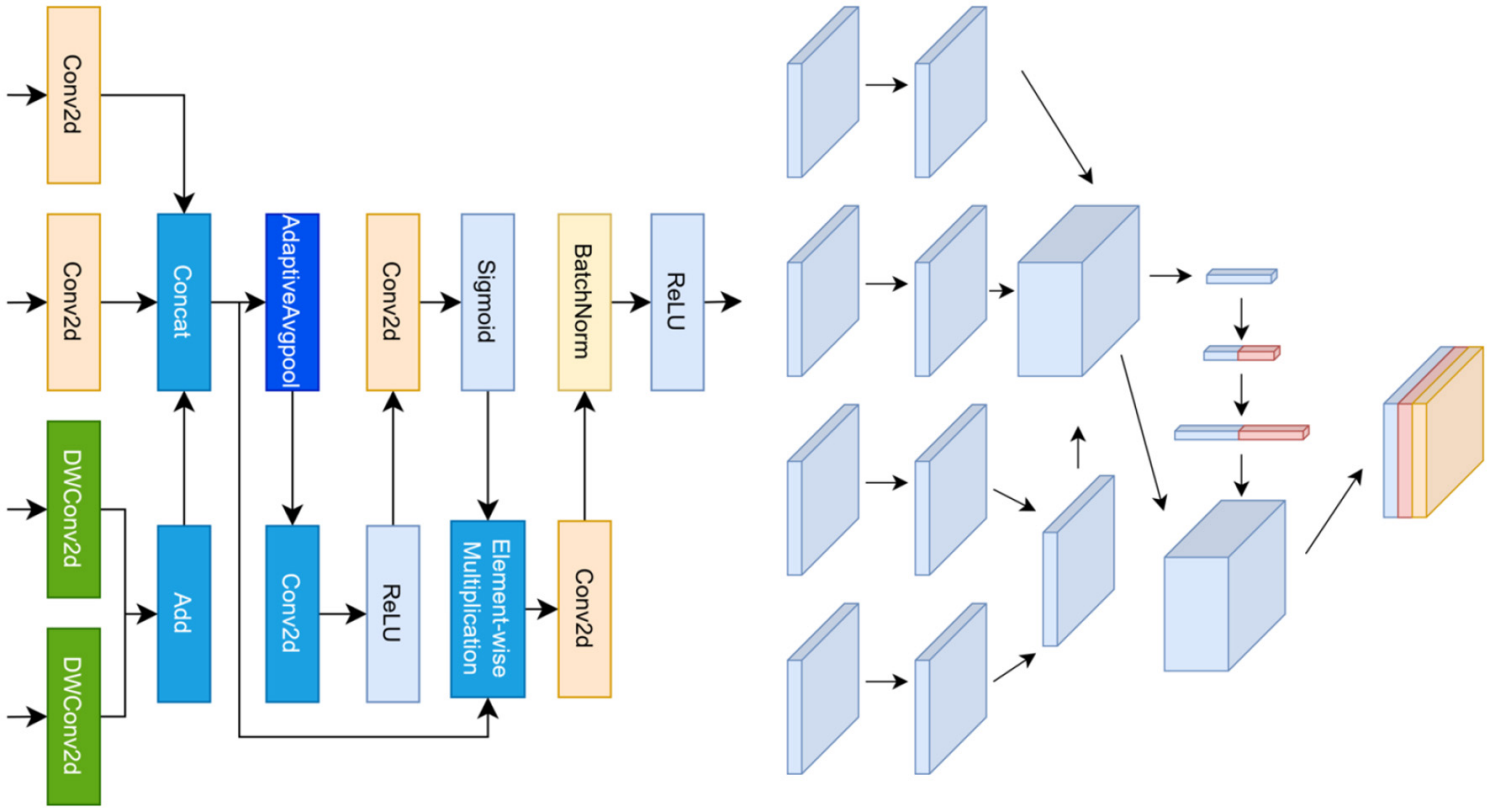
Architecture of the Dual-Path Differential Feature (DPF) module. **Left**: the input feature map is decomposed into a detail path using depthwise convolutions and a context path using dilated convolutions, enabling explicit modeling of high- and low-frequency components. Differential enhancement is computed through a learnable projection followed by residual modulation. **Right**: channel-wise feature transformations illustrate how DPF amplifies structural transitions while suppressing homogeneous background responses, yielding contrast-enhanced representations for downstream decoding.

Conventional convolutional operations tend to entangle high-frequency structural details with low-frequency contextual patterns, making feature representations susceptible to contrast attenuation under speckle noise and iso-echoic conditions. As a result, diagnostically relevant structural variations may be suppressed by dominant homogeneous background responses. To explicitly enhance discriminative structural transitions under such conditions, we introduce the **Dual-Path Differential Feature (DPF)** module. An overview of the DPF architecture is illustrated in Figure 3.

#### 3.3.1 Dual-path decomposition

Given an encoder feature map *F*∈ℝ^*C*×*H*×*W*^, the DPF module decomposes it into a high-frequency *detail path* *F*_*d*_ and a low-frequency *context path* *F*_*c*_. The detail path employs depthwise convolutions to capture localized structural variations, while the context path utilizes dilated convolutions to model broader contextual information:


$$F_{d}=\phi \left(\text{DWConv}\left(F\right)\right),\;F_{c}=\phi \left(\text{DilatedConv}\left(F\right)\right),$$

where ϕ(·) denotes batch-normalized ReLU activation. This decomposition aligns with the characteristics of ultrasound imaging, in which diagnostically meaningful structures often manifest as localized high-frequency responses superimposed on slowly varying parenchymal textures.

#### 3.3.2 Differential feature enhancement

Based on the decomposed representations, an explicit differential response is computed as:


$$F_{\text{diff}}=\sigma \left(W_{d}*\left(F_{d}-F_{c}\right)\right),$$

where *W*_*d*_ denotes a 1 × 1 learnable projection and σ(·) represents sigmoid normalization. Larger responses correspond to pronounced local structural transitions, whereas homogeneous regions yield smaller values, enabling selective emphasis of informative structures under low-contrast and noise-corrupted conditions.

#### 3.3.3 Residual modulation

The enhanced representation is obtained via residual modulation:


$$F_{\text{DPF}}=F+\alpha F_{\text{diff}},$$

where α is a learnable scalar controlling the contribution of the differential response. In addition, a channel-wise modulation mechanism is employed to further suppress noise-amplifying channels, thereby improving robustness to speckle interference and local artifacts.

#### 3.3.4 Distinction from existing modules

Unlike reweighting-based attention mechanisms such as SE and CBAM (13, 14), which primarily adjust the importance of existing feature responses, the proposed DPF module performs an explicit and learnable high–low frequency decomposition to generate new contrast-enhanced representations. This differential modeling enables more effective separation of informative structural variations from homogeneous background patterns, which is particularly advantageous for thyroid ultrasound images characterized by low contrast and heterogeneous echogenicity.

### 3.4 Edge-Aware ASPP-E module

**Figure 4:**
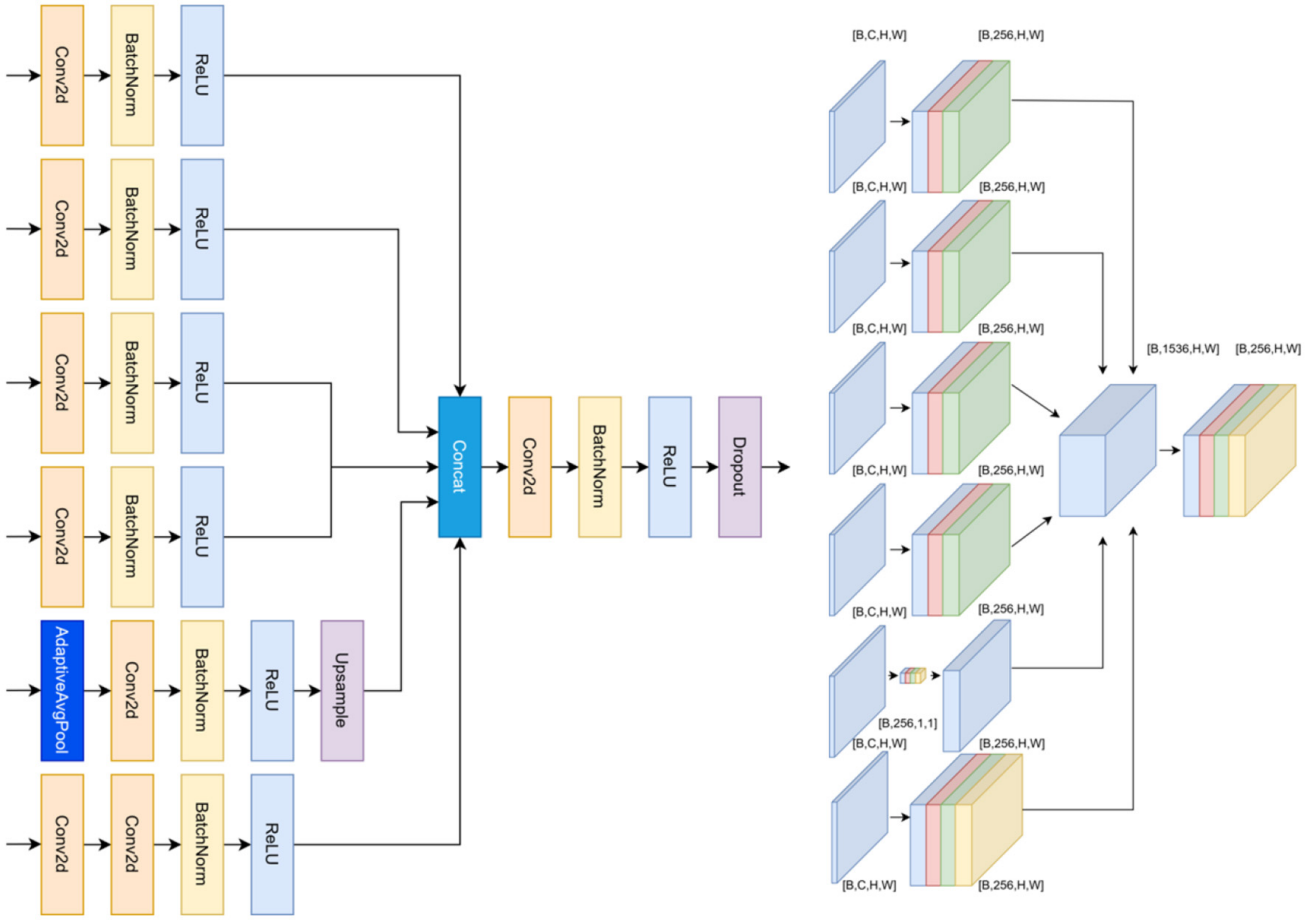
Architecture of the Edge-Aware ASPP-E module. **Left**: ASPP-E extends classical ASPP by introducing a structure-aware modulation branch that predicts a spatial confidence map. Each dilation branch is adaptively modulated to enhance structurally informative responses while preserving multi-scale semantic context. **Right**: Feature-level interactions illustrate how structure-aware modulation strengthens subtle structural variations and suppresses homogeneous parenchymal regions under speckle degradation.

Thyroid nodules exhibit substantial variation in size, morphology, and internal structural appearance across patients and acquisition conditions. Effective multi-scale contextual modeling is therefore essential for robust ultrasound segmentation. However, conventional atrous spatial pyramid pooling (ASPP) (5) is not explicitly tailored to ultrasound-specific structural ambiguity and often produces over-smoothed representations under speckle noise or acoustic shadowing. To address this limitation, we introduce an **Edge-Aware Atrous Spatial Pyramid Pooling with Enhancement (ASPP-E)** module. An overview of the ASPP-E architecture is illustrated in Figure 4.

#### 3.4.1 Multi-scale feature extraction

Given an input feature map *F*_in_, ASPP-E applies a set of parallel dilated convolutions with dilation rates {1, 6, 12, 18}, together with an additional global pooling branch. These parallel branches capture contextual information at multiple receptive-field scales, enabling effective modeling of nodule shape variability and surrounding tissue context.

#### 3.4.2 Structure-aware modulation

To reinforce structurally informative responses, a learnable structure-aware predictor is introduced:


$$A_{\text{edge}}=\sigma \left(\text{Con}{\text{v}}_{1\times 1}\left(F_{\text{in}}\right)\right),$$

where σ(·) denotes sigmoid activation. The predicted map encodes the spatial confidence of edge- and structure-related cues. Each dilation branch output *F*_*r*_*i*__ is then adaptively modulated as


$${\tilde{F}}_{r_{i}}=F_{r_{i}}⊙A_{\text{edge}}.$$

This modulation suppresses homogeneous background activations while enhancing orientation-sensitive structural cues, thereby improving the consistency of multi-scale representations under low-contrast and noise-corrupted imaging conditions.

#### 3.4.3 Fusion and projection

All modulated multi-scale features are concatenated and subsequently compressed through a 1 × 1 convolution, yielding a structure-consistent multi-scale representation *F*_ASPP-E_ that is forwarded to the decoder.

#### 3.4.4 Distinction from classical ASPP

Unlike classical ASPP, which aggregates multi-scale features uniformly, ASPP-E incorporates explicit structure-aware reinforcement through adaptive modulation. This design enables more effective preservation of informative structural patterns while reducing the influence of speckle noise and shadow-induced artifacts commonly encountered in thyroid ultrasound imaging.

### 3.5 Edge-Guided Hybrid Decoder (EHD)

**Figure 5:**
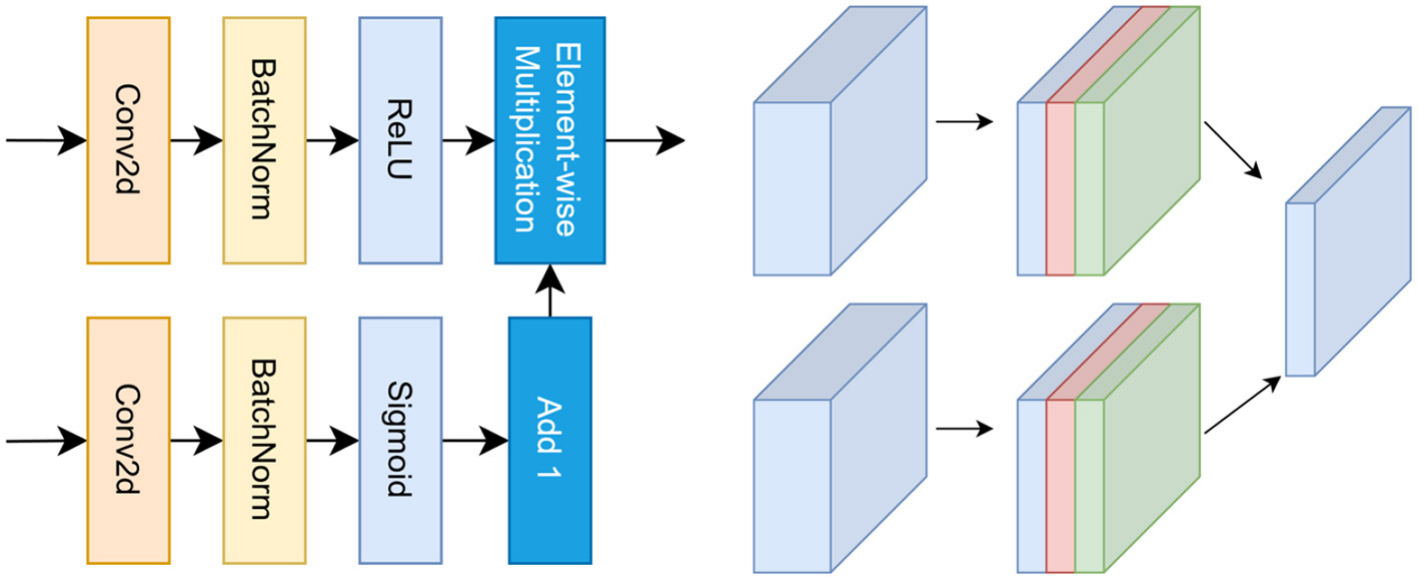
Architecture of the proposed Edge-Guided Hybrid Decoder (EHD). **Left**: EHD estimates a confidence map from deep semantic features and uses it to selectively gate shallow encoder features, suppressing noise-dominated activations before fusion. **Right**: The refined shallow features are merged with upsampled deep semantic representations through a hybrid convolutional block, enhancing structural detail preservation while preventing over-smoothing in speckle-degraded regions.

Shallow encoder features preserve fine spatial details but are highly sensitive to speckle noise and local artifacts. In U-Net–style decoders (4), direct skip connections often inject noise-dominated activations into the decoding process, which can destabilize feature fusion under adverse ultrasound imaging conditions such as speckle degradation and acoustic shadowing (3). To mitigate this issue, we propose an **Edge-Guided Hybrid Decoder (EHD)**, whose overall structure is shown in Figure 5.

#### 3.5.1 Edge confidence estimation

Given a deep semantic feature map *F*_sem_ and its corresponding shallow encoder feature *F*_shallow_, EHD first estimates a spatial confidence map:


$$A_{\text{conf}}=\sigma \left(\text{Con}{\text{v}}_{1\times 1}\left(F_{\text{sem}}\right)\right),$$

where σ(·) denotes sigmoid activation. This map encodes the reliability of shallow features at each spatial location.

#### 3.5.2 Selective shallow-feature injection

The shallow feature map is adaptively gated using the estimated confidence:


$${\tilde{F}}_{\text{shallow}}=A_{\text{conf}}⊙F_{\text{shallow}},$$

thereby suppressing noise-dominated responses before fusion with deep semantic features.

#### 3.5.3 Hybrid fusion with semantic context

The refined shallow features are fused with upsampled semantic representations through a hybrid convolutional block:


$$F_{\text{fuse}}=\phi \left(\text{Conv}\left(\left[{\tilde{F}}_{\text{shallow}},\text{Upsample}\left(F_{\text{sem}}\right)\right]\right)\right),$$

where ϕ(·) denotes batch-normalized ReLU activation. This hybrid fusion combines fine-grained spatial precision with robust semantic context, yielding more stable and structurally consistent decoding under noise-corrupted conditions.

#### 3.5.4 Distinction from U-Net-style skip connections

In contrast to U-Net–style decoders, which directly propagate shallow features without filtering (4), EHD employs confidence-guided gating to selectively inject reliable information into the decoder. This strategy reduces the propagation of noise-induced artifacts and improves boundary preservation in challenging ultrasound images (3, 10).

### 3.6 Loss function and optimization

Thyroid nodules typically occupy a small portion of ultrasound images and exhibit thin, irregular contours that are highly susceptible to speckle noise and acoustic shadowing. These characteristics result in pronounced class imbalance and structural ambiguity. To jointly encourage stable pixel-level optimization and robust region-level consistency, we adopt a hybrid loss function that combines pixel-wise cross-entropy with overlap-based losses, following a widely used paradigm in medical image segmentation.

Pixel-wise cross-entropy facilitates stable foreground–background discrimination across the entire field of view, while overlap-based losses explicitly optimize region similarity between predictions and ground-truth annotations. In particular, Dice and IoU losses emphasize global shape consistency rather than individual pixel accuracy, making them well suited for segmenting small or fragmented thyroid nodules under low-contrast and noisy imaging conditions.

To further emphasize diagnostically challenging regions, online hard example mining (OHEM) (21) is incorporated into the cross-entropy term. OHEM selectively backpropagates gradients from high-error pixels, thereby suppressing the dominance of easy background regions and encouraging the network to focus on noise-corrupted or structurally ambiguous areas, such as shadow-affected boundaries or iso-echoic nodules. This mechanism complements the proposed DPF, ASPP-E, and EHD modules by reinforcing learning on structural patterns that critically influence segmentation robustness.

Formally, the overall objective function is defined as a weighted combination of cross-entropy loss with OHEM and overlap-based losses. Network optimization is performed using stochastic gradient descent (SGD) with momentum, together with a warm-up phase and polynomial learning-rate decay (22). This optimization strategy promotes stable convergence during early training and enables effective fine-grained refinement in later stages, facilitating joint optimization of deep semantic representations and the proposed structure-aware components.

## 4 Experiments and results

### 4.1 Comparison with state-of-the-art methods

To evaluate the effectiveness of the proposed DPF–EHDNet, we compare it with nine representative semantic segmentation frameworks, including U-Net (4), DeepLabv3+ (5), DAC-Net (23), DC-Net (24), CRBNet (25), and TRFE-Net (26), as well as three additional state-of-the-art models, namely nnU-Net (6), TransUNet (27), and Swin-UNet (28).

These methods cover a broad range of architectural paradigms, including classical encoder–decoder structures, atrous convolution-based context modeling, refinement-oriented strategies, and recent Transformer-based approaches. This diverse selection provides a comprehensive and up-to-date benchmark for thyroid ultrasound segmentation.

The quantitative results in Table 2 indicate that DPF–EHDNet achieves the best overall performance across most evaluation metrics. In addition to region-based metrics (mIoU and Dice), boundary-sensitive metrics including HD95 and ASSD are also reported. The proposed method achieves lower HD95 and ASSD values, demonstrating its superior ability in preserving fine boundary structures.

**Table 2:** Quantitative comparison with state-of-the-art segmentation models, including CNN-based and Transformer-based methods, on the thyroid ultrasound dataset.

Method	mIoU(%)	Dice(%)	Prec.(%)	Rec.(%)	F1(%)	HD95(px)	ASSD	BIoU
U-Net (4)	76.02	86.38	85.20	87.59	86.38	16.57	6.37	0.34
DeepLabv3+ (5)	90.50	90.66	91.13	90.18	90.66	11.22	4.39	0.39
DAC-Net (23)	73.62	84.81	85.85	83.79	84.81	18.78	7.32	0.33
DC-Net (24)	75.51	84.38	81.77	91.27	84.97	16.60	5.52	0.39
CRBNet (25)	79.45	88.55	90.11	87.03	88.55	14.03	5.27	0.40
TRFE-Net (26)	81.46	89.78	89.39	90.18	89.78	12.02	4.42	0.47
nnU-Net (6)	77.99	86.18	86.44	89.06	86.18	34.31	10.63	0.45
TransUNet (27)	83.42	89.94	92.11	89.45	89.94	8.11	2.94	0.53
Swin-UNet (28)	77.61	85.17	85.56	88.79	85.17	32.31	9.87	0.43
**DPF–EHDNet (Ours)**	**93.09**	**93.35**	**93.98**	**92.73**	**93.35**	**7.64**	**2.84**	**0.56**

The bold values indicate the best performance among all compared methods in each table. Specifically, higher values are better for mIoU, Dice, Precision, Recall, F1, and BIoU, whereas lower values are better for HD95, ASSD, Params, and FLOPs. The bold values are used to highlight the superior performance of our proposed method or module.

Classical encoder–decoder architectures such as U-Net show limited performance on thyroid ultrasound images, with an mIoU of 76.02%. This result reflects the difficulty of handling modality-specific challenges, including speckle noise, heterogeneous echogenicity, and low lesion–background contrast, using direct skip connections without explicit noise suppression or structural modeling.

DAC-Net and DC-Net exhibit relatively lower segmentation accuracy compared with DeepLabv3+ and TRFE-Net. Although these methods introduce refinement or context modeling mechanisms, their performance suggests that global or lightweight aggregation alone may be insufficient to robustly capture heterogeneous structural patterns commonly observed in thyroid ultrasound images.

DeepLabv3+ achieves stronger results owing to its atrous spatial pyramid pooling design, which effectively captures multi-scale contextual information. However, its representations do not explicitly model structural ambiguity caused by iso-echoic nodules or shadow-induced distortions.

In contrast, DPF–EHDNet yields an improvement of 2.59% in mIoU over DeepLabv3+, together with consistent gains in precision, recall, and F1-score, indicating improved region-level consistency under challenging imaging conditions.

Furthermore, recently proposed advanced segmentation models, including nnU-Net, TransUNet, and Swin-UNet, are incorporated for comparison. nnU-Net demonstrates strong adaptability to medical datasets, while TransUNet and Swin-UNet leverage Transformer architectures to enhance global contextual modeling. However, their performance is still inferior to the proposed DPF–EHDNet, particularly in boundary-sensitive metrics such as HD95 and ASSD. This suggests that, despite improved global representation, these methods may struggle to accurately delineate fine-grained lesion boundaries in thyroid ultrasound images. In contrast, the proposed method achieves superior performance by explicitly modeling structural details and boundary information.

**Figure 6:**
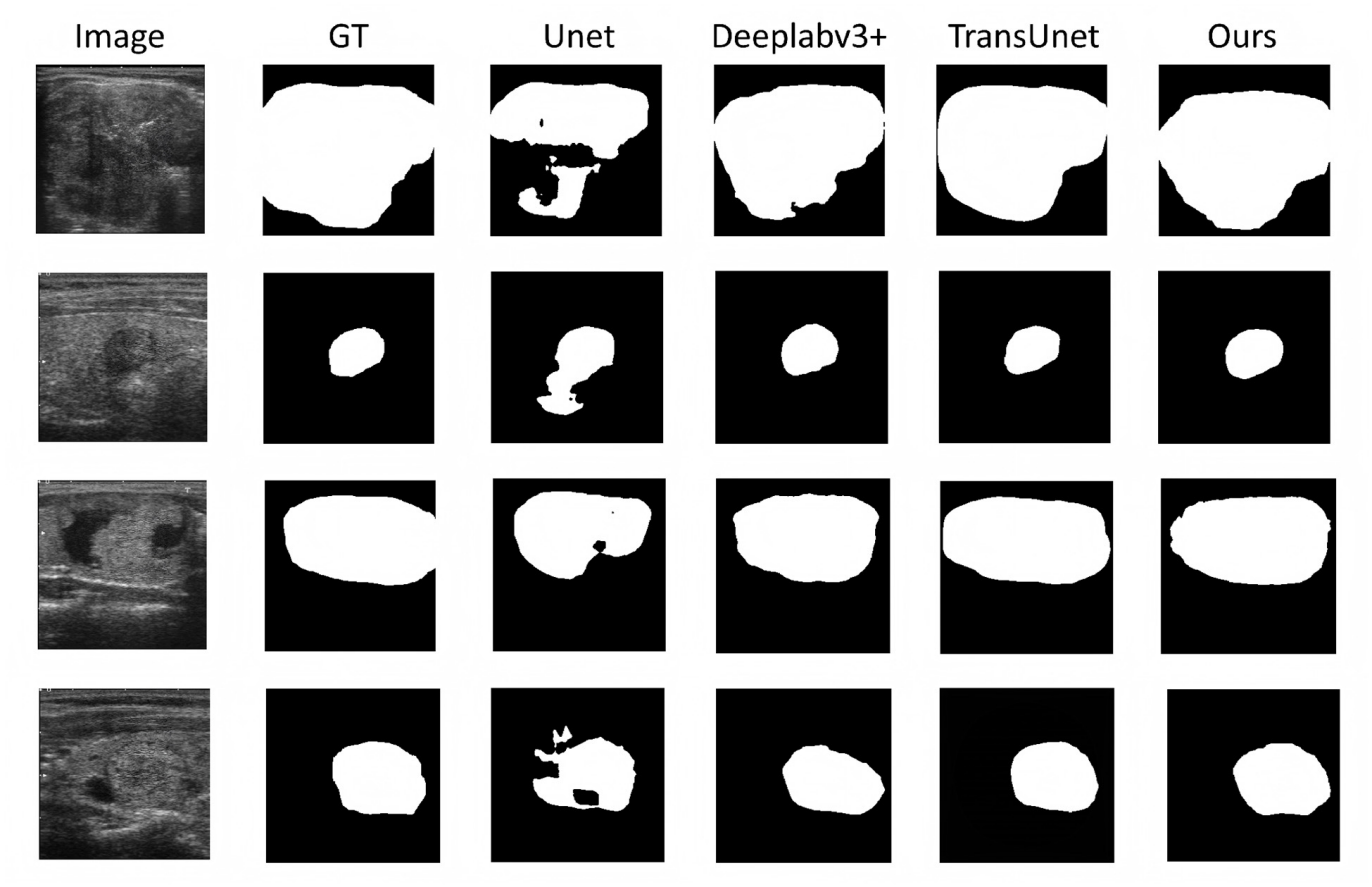
Qualitative comparison of thyroid ultrasound segmentation results on representative test samples. From top to bottom, each row shows the original ultrasound image, the ground truth annotation, and the segmentation results produced by U-Net, DeepLabV3+, TransUNet, and the proposed DPF–EHDNet, respectively. Each column corresponds to a different test case. The proposed method produces more contiguous lesion regions and preserves irregular structural details more consistently, particularly for nodules with weak contrast, heterogeneous appearance, and speckle noise interference.

The qualitative comparisons in Figure 6 are consistent with the quantitative results. U-Net frequently generates fragmented lesion regions and false-positive responses in homogeneous parenchyma, while DeepLabv3+ improves overall shape consistency but tends to produce over-smoothed boundaries in low-contrast regions. TransUNet further enhances global contextual modeling, yet still exhibits boundary inaccuracies and occasional shape distortions.

These observations are further supported by boundary-sensitive metrics (HD95 and ASSD), where competing methods exhibit larger boundary deviations. In contrast, the proposed method achieves consistently lower boundary errors, demonstrating its effectiveness in preserving fine structural details and edge information.

**Figure 7:**
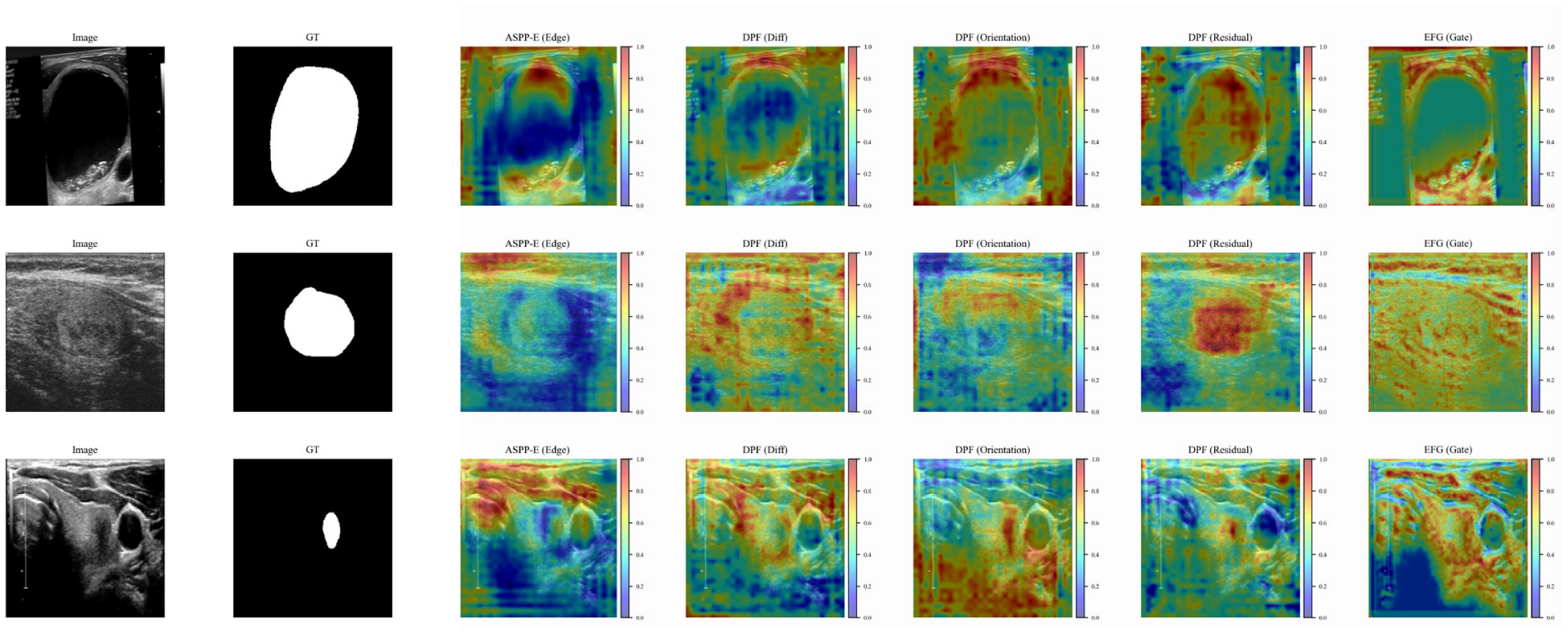
Visualization of intermediate feature responses of different modules, including ASPP-E, DPF, and EHD. The proposed modules exhibit enhanced sensitivity to structural and boundary-related regions, highlighting their ability to capture edge-aware and context-aware representations.

**Figure 8:**
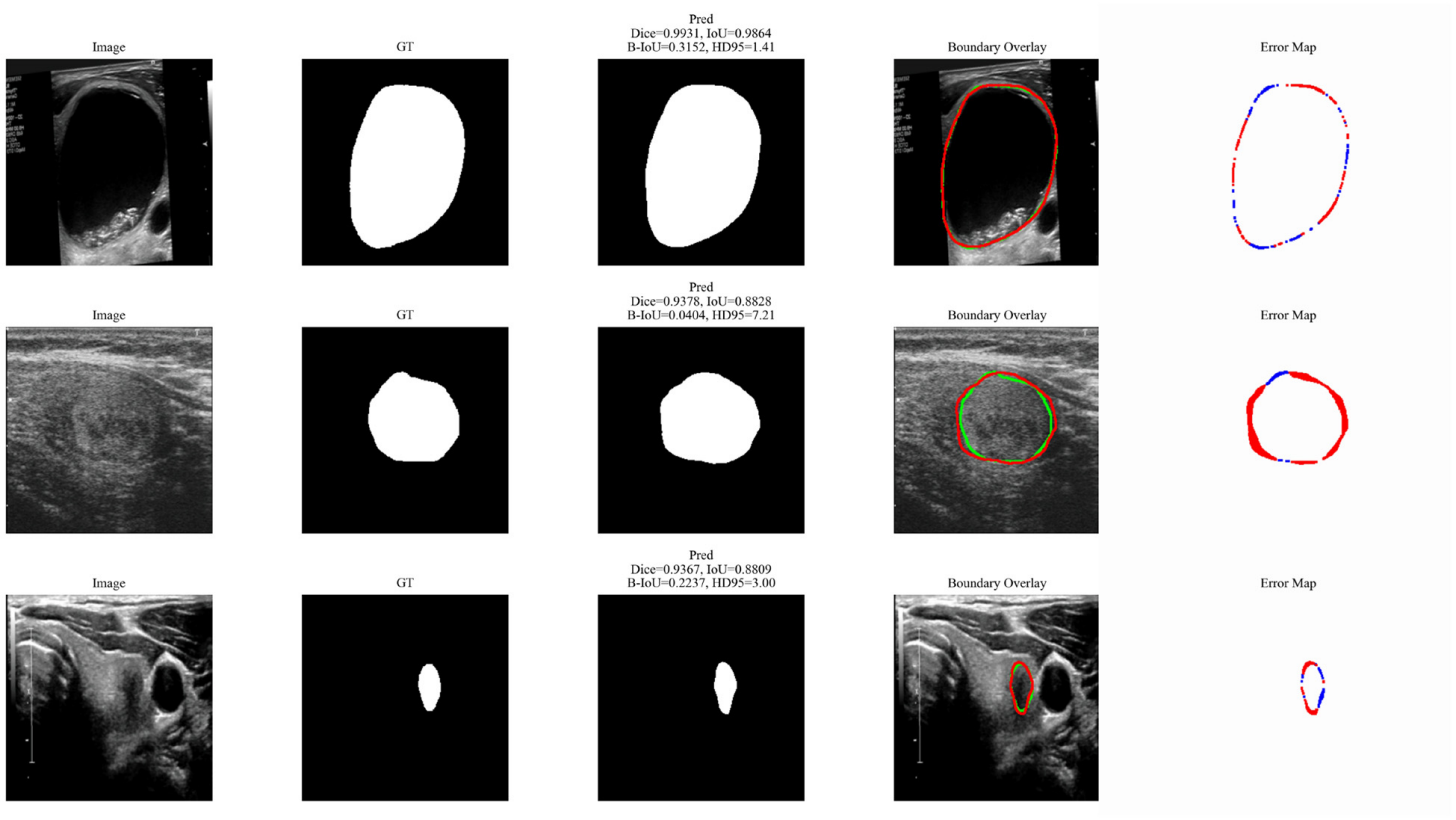
Visualization of boundary overlays and error maps for representative test samples. The green and red contours denote the predicted and ground-truth boundaries, respectively. The error maps highlight boundary deviations. The proposed method produces more accurate and smoother boundaries with fewer errors, demonstrating improved boundary delineation and structural consistency.

Furthermore, feature map visualizations in Figure 7 and boundary error analyses in Figure 8 further confirm that the proposed modules effectively enhance structural representation and boundary delineation.

In comparison, DPF–EHDNet yields more coherent lesion masks and better preserves irregular structural details across diverse test cases. These visual observations align with the improvements in mIoU, Dice, and balanced precision–recall performance reported in Table 2, suggesting enhanced segmentation stability under noise-corrupted ultrasound conditions.

Overall, the experimental results demonstrate that the proposed DPF–EHDNet consistently outperforms both conventional CNN-based and recent Transformer-based segmentation methods across multiple evaluation metrics. These findings further validate the effectiveness of incorporating differential feature modeling and boundary-aware mechanisms for robust thyroid ultrasound segmentation.

### 4.2 Computational complexity analysis

To further evaluate the computational efficiency of the proposed method, we compare the model complexity in terms of the number of parameters (Params) and floating-point operations (FLOPs), as summarized in Table 3.

**Table 3:** Computational complexity comparison of different segmentation models, including parameter count (Params) and floating-point operations (FLOPs).

Method	Params (M)	FLOPs (G)
U-Net	31.04	54.66
DeepLabv3+	58.75	19.82
DAC-Net	1.28	0.70
DC-Net	54.41	26.83
CRBNet	1.81	2.98
TRFE-Net	44.90	92.90
nnU-Net	30.10	71.90
TransUNet	93.23	32.24
Swin-UNet	27.15	5.92
**DPF–EHDNet (Ours)**	**66.11**	**24.32**

The bold values indicate the best performance among all compared methods in each table. Specifically, higher values are better for mIoU, Dice, Precision, Recall, F1, and BIoU, whereas lower values are better for HD95, ASSD, Params, and FLOPs. The bold values are used to highlight the superior performance of our proposed method or module.

As shown in Table 3, the proposed DPF–EHDNet achieves a favorable balance between segmentation performance and computational cost. Specifically, the parameter count of DPF–EHDNet is comparable to commonly used architectures such as DeepLabv3+ and DC-Net, while remaining significantly lower in parameter count than more complex models such as TransUNet.

In terms of computational cost, the FLOPs of DPF–EHDNet are lower than several high-capacity models, including TRFE-Net and nnU-Net, indicating improved efficiency in feature extraction and decoding processes. Although lightweight models such as DAC-Net and CRBNet require fewer parameters and FLOPs, their segmentation performance is notably inferior, suggesting a trade-off between model capacity and accuracy.

Overall, these results demonstrate that the proposed DPF–EHDNet maintains strong segmentation performance without introducing excessive computational overhead, achieving a favorable trade-off between accuracy and efficiency. This highlights the potential of the proposed method for practical deployment in clinical ultrasound image analysis.

### 4.3 Robustness analysis under different random seeds

To evaluate the robustness of the proposed framework with respect to stochastic optimization, DPF–EHDNet was trained and evaluated using three different random seeds while keeping the network architecture, data split, and all hyperparameter settings unchanged. The quantitative results are summarized in Table 4.

**Table 4:** Robustness analysis of DPF–EHDNet under different random seeds.

Seed	mIoU(%)	Dice(%)	Precision(%)	Recall(%)	F1(%)
10	93.02	93.28	93.93	92.64	93.28
100	93.00	93.26	94.05	92.48	93.26
1,000	93.07	93.33	94.00	92.66	93.33

As shown in Table 4, the proposed method exhibits stable performance across different random seeds. Variations in mIoU, Dice, precision, recall, and F1-score remain marginal, indicating that the optimization process is not sensitive to random initialization. These results suggest that DPF–EHDNet achieves consistent segmentation performance under stochastic training conditions.

### 4.4 Ablation study on module combinations

To evaluate the individual contributions and complementary effects of the proposed modules, we conduct ablation experiments by selectively enabling the Dual-Path Differential Feature (DPF), ASPP-E, and Edge-Guided Hybrid Decoder (EHD).

As shown in Table 5, each module individually improves segmentation performance over the baseline, with consistent gains observed across all evaluation metrics. Although improvements in region-based metrics such as mIoU and Dice are relatively modest, this is expected as performance approaches saturation.

**Table 5:** Ablation study on the complementary effects of ASPP-E, DPF, and EHD modules.

Method	mIoU(%)	Dice(%)	Prec.(%)	Rec.(%)	F1(%)	HD95(px)	ASSD	BIoU
ASPP-E	92.91	93.18	93.62	92.73	93.18	8.12	3.08	0.54
DPF	92.88	93.16	93.49	92.81	93.16	8.20	3.15	0.53
EHD	92.86	93.11	93.85	92.39	93.11	8.24	3.18	0.52
ASPP-E + DPF	92.96	93.21	93.77	92.65	93.21	8.05	3.02	0.55
DPF + EHD	92.92	93.15	93.79	92.49	93.15	7.88	2.96	0.55
ASPP-E + EHD	92.94	93.19	93.67	92.71	93.19	8.08	3.04	0.54

Each module is evaluated individually and in pairwise combinations.

We further analyze boundary-sensitive metrics, including HD95, ASSD, and Boundary IoU. All three modules consistently reduce HD95 and ASSD while increasing Boundary IoU, indicating improved boundary localization and structural consistency.

Among the individual components, ASPP-E primarily enhances multi-scale structural representation, DPF improves modeling of structural transitions, and EHD strengthens boundary refinement during decoding. For pairwise combinations, ASPP-E+DPF achieves the highest region-based performance, while DPF+EHD shows stronger boundary accuracy, suggesting that different module combinations emphasize complementary aspects of segmentation.

Overall, combining all three modules yields the most balanced performance across both region and boundary metrics, demonstrating their complementary roles in the proposed framework.

### 4.5 Ablation study on differential feature enhancement (DPF)

To examine the contribution of explicit differential feature modeling, the proposed DPF module was compared with four representative enhancement mechanisms, including SE (13), CBAM (14), NSBR (29), and LKA (30). These methods represent channel recalibration, joint spatial–channel attention, local structural refinement, and enlarged receptive-field modeling, respectively. Quantitative results are summarized in Table 6.

**Table 6:** Ablation study on differential feature enhancement modules.

Method	mIoU(%)	Dice(%)	Prec.(%)	Rec.(%)	F1(%)	HD95(px)	ASSD	BIoU
SE (13)	92.78	93.03	93.79	92.29	93.03	8.40	3.30	0.48
CBAM (14)	92.92	93.18	93.71	92.47	93.17	8.62	3.60	0.49
NSBR (29)	92.77	93.01	93.62	92.42	93.02	8.45	3.32	0.48
LKA (30)	92.97	93.22	93.87	92.44	93.22	8.58	3.55	0.48
**DPF (Ours)**	**92.98**	**93.23**	**93.93**	**92.57**	**93.24**	**8.20**	**3.15**	**0.53**

The bold values indicate the best performance among all compared methods in each table. Specifically, higher values are better for mIoU, Dice, Precision, Recall, F1, and BIoU, whereas lower values are better for HD95, ASSD, Params, and FLOPs. The bold values are used to highlight the superior performance of our proposed method or module.

As shown in Table 6, the proposed DPF module achieves the best overall performance across all evaluation metrics. While the improvements in region-based metrics such as mIoU and Dice are relatively small, DPF consistently improves boundary-related measures, achieving lower HD95 and ASSD values and a higher Boundary IoU.

Compared with existing enhancement mechanisms, SE and CBAM mainly rely on global feature recalibration through channel or spatial attention, which may overlook subtle local structural variations. NSBR enhances local responses but lacks explicit modeling of feature transitions, while LKA emphasizes large receptive fields and global context continuity, with limited sensitivity to boundary details.

In contrast, DPF explicitly models differential feature responses between lesion and background regions, emphasizing structural transitions that are critical for accurate boundary delineation. This targeted modeling enables better capture of fine-grained structural variations and contributes to more consistent segmentation performance.

Although the numerical differences are relatively small, the improvements are consistent across all metrics and align with observable gains in boundary-related measures, indicating stable performance improvements rather than random variations.

### 4.6 Ablation study on the ASPP-E module

To evaluate the effectiveness of the proposed ASPP-E module, it was replaced with several representative multi-scale context aggregation strategies while keeping DPF and EHD unchanged. The compared modules include the classical ASPP from DeepLabv3+ (5), the Pyramid Pooling Module (PPM) from PSPNet (22), the lightweight ELPPM module (31), and the Cascade Attention Dense Field (CADF) module (32). The quantitative results are reported in Table 7.

**Table 7:** Ablation study on ASPP-E replacements with DPF and EHD fixed.

Method	mIoU(%)	Dice(%)	Prec.(%)	Rec.(%)	F1(%)	HD95(px)	ASSD	BIoU
ASPP (5)	92.65	92.89	92.91	92.44	92.89	8.65	3.42	0.43
CADF (32)	92.75	92.99	93.41	92.59	92.99	8.48	3.30	0.45
ELPPM (31)	92.77	93.02	93.46	92.59	93.03	8.72	3.48	0.42
PPM (22)	92.86	93.11	93.61	92.26	93.11	8.60	3.40	0.44
**ASPP-E (Ours)**	**92.94**	**93.20**	**93.64**	**92.76**	**93.20**	**8.12**	**3.08**	**0.54**

The bold values indicate the best performance among all compared methods in each table. Specifically, higher values are better for mIoU, Dice, Precision, Recall, F1, and BIoU, whereas lower values are better for HD95, ASSD, Params, and FLOPs. The bold values are used to highlight the superior performance of our proposed method or module.

The results indicate that ASPP-E provides consistent improvements over classical and attention-based pyramid structures. While ASPP and PPM effectively capture semantic context at multiple scales, they rely on fixed pooling or dilation configurations, which may limit their ability to adapt to complex structural variations.

Compared with these methods, ASPP-E achieves higher mIoU and Dice scores while also obtaining lower HD95 and ASSD values and a higher Boundary IoU, indicating improved structural consistency and more accurate boundary delineation. ELPPM and CADF introduce lightweight or attention-based enhancements, but may smooth out fine structural details in regions with heterogeneous echogenicity.

By incorporating structure-aware modulation into each dilation branch, ASPP-E enables adaptive emphasis on discriminative structural patterns across different receptive fields, leading to more stable multi-scale representations and improved segmentation performance under speckle noise and low-contrast conditions.

### 4.7 Ablation study on the EHD module

To further assess the effectiveness of the proposed Edge-Guided Hybrid Decoder (EHD), its fusion mechanism was replaced with several representative decoder strategies while keeping ASPP-E and DPF unchanged. The compared methods include the Attention Gate (AG) from U-Net++ (10), CBAM applied to skip connections (CBAM-skip) (33), the Bottleneck Attention Module (BAM) (34), and a hybrid CBAM–AG fusion block (35). Quantitative results are summarized in Table 8.

**Table 8:** Ablation study on decoder fusion strategies with ASPP-E and DPF fixed.

Method	mIoU(%)	Dice(%)	Prec.(%)	Rec.(%)	F1(%)	HD95(px)	ASSD	BIoU
AG (10)	92.84	93.09	93.65	92.28	93.09	8.38	3.32	0.47
CBAM-skip (33)	92.79	93.05	93.66	92.22	93.05	8.42	3.36	0.48
BAM (34)	92.87	93.14	93.65	92.60	93.13	8.36	3.40	0.48
CBAM–AG (35)	92.87	93.12	93.68	92.58	93.13	8.40	3.38	0.48
**EHD (Ours)**	**92.89**	**93.15**	**93.69**	**92.61**	**93.15**	**8.24**	**3.18**	**0.52**

The bold values indicate the best performance among all compared methods in each table. Specifically, higher values are better for mIoU, Dice, Precision, Recall, F1, and BIoU, whereas lower values are better for HD95, ASSD, Params, and FLOPs. The bold values are used to highlight the superior performance of our proposed method or module.

All attention-based decoder variants improve segmentation accuracy relative to unfiltered skip connections, highlighting the importance of selectively modulating shallow features during decoding. However, these generic mechanisms primarily rely on feature saliency and do not explicitly account for structural reliability under ultrasound-specific degradations.

Compared with these methods, EHD achieves higher mIoU and Dice scores while also obtaining lower HD95 and ASSD values and a higher Boundary IoU, indicating improved structural consistency and more accurate alignment between predicted regions and lesion boundaries.

In contrast, the proposed EHD leverages confidence cues derived from deep semantic features to guide shallow feature fusion. This confidence-guided strategy enables more effective integration of semantic and structural information, yielding more coherent lesion masks and consistent gains across all evaluation metrics while maintaining a compact decoder structure.

### 4.8 Cross-dataset robustness and transferability evaluation

To comprehensively evaluate the robustness and cross-dataset transferability of the proposed framework under a consistent training protocol, we conduct both intra-dataset and cross-dataset experiments on multiple public datasets, including a brain MRI tumor segmentation dataset (19), a breast ultrasound dataset (20), and thyroid ultrasound datasets (TN3K and DDTI).

For intra-dataset evaluation, the model is trained and tested on each dataset independently using the official splits, while maintaining the same network architecture, loss configuration, and optimization strategy as those used in the thyroid ultrasound experiments. In addition, to further assess generalization ability under domain shift, we perform a strict cross-dataset experiment by training the model on TN3K and directly testing it on DDTI without any fine-tuning. This setting is widely adopted in medical image segmentation to evaluate cross-dataset generalization under limited annotation availability, and provides a realistic assessment of model transferability across clinical datasets.

Quantitative results for both intra-dataset and cross-dataset evaluations are summarized in Table 9.

**Table 9:** Cross-dataset robustness and transferability evaluation of DPF–EHDNet.

Dataset/Setting	mIoU(%)	Dice(%)	Precision(%)	Recall(%)	F1(%)
Brain MRI (intra)	91.36	90.67	90.25	91.07	90.66
Breast US (intra)	79.18	76.36	84.74	69.50	76.36
COCO 2017 (intra)	82.08	86.37	86.63	86.12	86.37
TN3K → DDTI (cross)	80.36	78.52	75.38	80.95	78.52

The proposed framework achieves strong performance on the brain MRI dataset, demonstrating robustness under substantially different imaging modalities and tissue contrast characteristics. On the breast ultrasound dataset, which shares modality-specific challenges such as speckle noise and boundary ambiguity, DPF–EHDNet still maintains favorable segmentation accuracy. However, the recall on this dataset is relatively lower than that on the thyroid ultrasound benchmark, suggesting that some lesion regions may remain under-segmented under severe boundary ambiguity or heterogeneous appearance. This performance gap is likely related to differences in lesion morphology, annotation characteristics, and data distribution between thyroid and breast ultrasound images, and further indicates that cross-organ ultrasound transfer remains challenging despite the shared imaging modality.

To further validate cross-dataset generalization, the model trained on TN3K is directly evaluated on DDTI without any fine-tuning. Despite the domain gap between different thyroid ultrasound datasets, the model maintains reasonable performance, demonstrating its robustness to variations in data distribution and acquisition conditions.

Performance on the COCO subset reflects the model's behavior under extreme domain shifts between natural images and medical ultrasound data. This experiment serves as a stress test for architectural stability rather than a clinically relevant evaluation.

Overall, the results demonstrate that DPF–EHDNet achieves stable and reliable performance across both intra-dataset and cross-dataset settings, highlighting its robustness to domain shifts and strong generalization capability in medical image segmentation tasks.

### 4.9 Comparison with traditional segmentation methods

To further validate the effectiveness of the proposed method, we compared DPF–EHDNet with several traditional segmentation approaches commonly used in clinical practice, including Otsu thresholding, Active Contour (Snake), and Region Growing.

As shown in Table 10, traditional methods exhibit inferior performance across all evaluation metrics. Otsu thresholding is highly sensitive to intensity variations and fails to accurately delineate lesion boundaries in low-contrast ultrasound images. Region Growing suffers from noise sensitivity and often leads to incomplete or fragmented segmentation results. Active Contour improves contour smoothness but still struggles with weak boundaries and heterogeneous regions.

**Table 10:** Comparison with traditional segmentation methods.

Method	mIoU(%)	Dice(%)	Prec.(%)	Rec.(%)	F1(%)	HD95(px)	ASSD	BIoU
Otsu Thresholding	23.63	11.81	8.12	48.17	11.81	267.98	89.61	0.023
Active Contour	55.16	33.19	38.11	50.49	33.19	127.06	57.96	0.021
Region Growing	40.30	8.92	21.91	8.90	8.92	261.22	117.77	0.010
**DPF–EHDNet (Ours)**	**93.09**	**93.35**	**93.98**	**92.73**	**93.35**	**7.64**	**2.84**	**0.56**

The bold values indicate the best performance among all compared methods in each table. Specifically, higher values are better for mIoU, Dice, Precision, Recall, F1, and BIoU, whereas lower values are better for HD95, ASSD, Params, and FLOPs. The bold values are used to highlight the superior performance of our proposed method or module.

In contrast, the proposed DPF–EHDNet achieves substantially higher segmentation accuracy and significantly lower boundary errors (HD95 and ASSD), demonstrating its superiority in capturing complex structural patterns and handling noise-corrupted ultrasound data. These results highlight the limitations of conventional methods and emphasize the necessity of advanced deep learning-based approaches for robust thyroid ultrasound segmentation.

## 5 Discussion and conclusion

### 5.1 Discussion

#### 5.1.1 Impact of thyroid-oriented feature modeling

This study proposes DPF–EHDNet, a segmentation framework specifically designed to address the characteristic degradations of thyroid ultrasound imaging. Across all experiments, the proposed method achieves consistently improved segmentation performance, as reflected by higher mIoU, Dice, precision, recall, and F1-score. These improvements suggest that explicitly modeling ultrasound-specific structural characteristics contributes to enhanced robustness against speckle noise, heterogeneous echogenicity, and low lesion–background contrast.

Importantly, the performance gains are not limited to a single dataset. The proposed framework maintains stable behavior in cross-dataset evaluations, indicating favorable generalization under varying imaging conditions and acquisition protocols. This observation supports the effectiveness of incorporating differential structural modeling and structure-aware decoding for ultrasound segmentation tasks, where conventional convolutional representations often struggle.

#### 5.1.2 Complementary roles of the proposed modules

The ablation results demonstrate that the three proposed modules play complementary roles within the overall architecture. ASPP-E primarily enhances multi-scale semantic representation, providing robust contextual information across varying lesion sizes and shapes. In contrast, DPF focuses on emphasizing differential structural transitions between nodules and surrounding tissue, while EHD selectively integrates reliable shallow features during decoding to mitigate noise propagation.

Their joint integration enables the network to simultaneously resolve coarse-scale contextual ambiguity and preserve fine-grained structural details. This modular design aligns well with the imaging characteristics of thyroid ultrasound, where diagnostic structures are often subtle, spatially irregular, and easily obscured by speckle interference. The results indicate that combining structure-aware modeling at both encoding and decoding stages is beneficial for achieving stable and reliable segmentation.

#### 5.1.3 Positioning relative to existing methods

Compared with representative state-of-the-art segmentation frameworks, including U-Net, DeepLabv3+, DAC-Net, DC-Net, CRBNet, and TRFE-Net, DPF–EHDNet consistently achieves superior performance across all evaluation metrics. While existing methods incorporate attention mechanisms, residual refinement, or multi-scale context aggregation, they typically rely on generic feature weighting strategies that do not explicitly account for ultrasound-specific structural reliability.

The proposed framework differs in that it integrates differential feature enhancement, structure-aware multi-scale modeling, and confidence-guided decoding within a unified architecture. This targeted design results in improved region-level consistency and enhanced robustness under noise-corrupted and low-contrast conditions, which are central challenges in thyroid ultrasound segmentation.

#### 5.1.4 Limitations and future directions

Despite the encouraging results, several limitations should be acknowledged. First, although multiple public datasets were incorporated, the current evaluation remains limited by the availability and diversity of annotated thyroid ultrasound data. In addition, although the hybrid dataset combines DDTI, TN-SCUI2020, and TN3K, it may still not fully capture the variability encountered in real clinical environments. Differences in ultrasound devices, operator expertise, and patient demographics can significantly affect image quality and lesion appearance. Future studies will include multi-institutional datasets to further assess robustness under diverse scanners, operators, and acquisition protocols.

Second, extremely faint nodules or lesions affected by severe acoustic shadowing remain challenging. Incorporating physics-informed priors, uncertainty modeling, or advanced augmentation strategies may further improve performance in these cases.

Third, while the proposed framework demonstrates strong segmentation accuracy, its deployment in real-time or resource-constrained clinical environments may still be limited by computational efficiency and model complexity. In particular, the integration of multiple functional modules increases the overall model complexity, which may pose challenges for real-time inference and hardware-constrained settings. Therefore, further optimization and lightweight design are necessary to facilitate practical clinical deployment.

Another limitation lies in the potential vulnerability of deep learning models to adversarial perturbations. Although this study focuses on improving structural representation and boundary delineation in thyroid ultrasound images, the robustness of the proposed model against adversarial manipulations or intentionally perturbed inputs has not been explicitly evaluated.

In clinical scenarios, variations in acquisition conditions, noise patterns, or subtle perturbations may affect model reliability. Therefore, future work will explore robustness enhancement strategies, such as adversarial training, uncertainty modeling, and domain generalization techniques, to further improve the stability and safety of the proposed framework in real-world clinical applications.

Future work will also explore extending the proposed design to other ultrasound applications and related imaging modalities, such as breast or vascular ultrasound, to further evaluate its adaptability and clinical utility.

### 5.2 Conclusion

In this study, we introduced DPF–EHDNet, a modular segmentation framework tailored to the specific characteristics of thyroid ultrasound imaging. By integrating differential feature enhancement, structure-aware multi-scale context modeling, and confidence-guided decoder fusion, the proposed method achieves consistent improvements over state-of-the-art architectures across multiple evaluation metrics.

The results demonstrate enhanced segmentation stability, improved region-level reliability, and favorable generalization across independently trained datasets. With further validation and optimization, DPF–EHDNet provides a promising methodological foundation for robust automated thyroid nodule segmentation and has potential to support computer-assisted diagnosis in clinical ultrasound workflows.

## Data Availability

Publicly available datasets were analyzed in this study. This data can be found here: The datasets analyzed in this study are publicly available from their original sources as cited in the manuscript. Access information and dataset descriptions are provided in the corresponding references.
